# Aminophylline Dosage In Asthma Exacerbations in Children: A Systematic Review

**DOI:** 10.1371/journal.pone.0159965

**Published:** 2016-08-02

**Authors:** Lewis Cooney, Ian Sinha, Daniel Hawcutt

**Affiliations:** 1 Department of Women's and Children's Health, Institute of Translational Medicine, University of Liverpool, Liverpool, Merseyside, United Kingdom; 2 National Institute for Health Research Alder Hey Clinical Research Facility, Alder Hey Children’s Hospital, Liverpool, Merseyside, United Kingdom; University of Rochester Medical Center, UNITED STATES

## Abstract

**Background:**

Adequate asthma treatment of childhood exacerbations with IV aminophylline depends on appropriate dosage. Recommendations to aim for a target therapeutic range may be inappropriate as serum concentrations correlate poorly with clinical improvement. This review aims to evaluate the evidence for the optimum dosage strategy of intravenous aminophylline in children suffering an exacerbation of asthma.

**Methods:**

A systematic review comparing dosage regimens of intravenous aminophylline in children suffering an exacerbation of asthma. Primary outcomes were time until resolution of symptoms, mortality and need for mechanical ventilation. Secondary outcomes were date until discharge criteria are met, actual discharge and adverse effects.

**Data sources:**

CENTRAL, CINAHL, MEDLINE and Web of Science. Search performed in March 2016

**Eligibility criteria:**

Studies using intravenous aminophylline in children with an acute exacerbation of asthma which reported the dosage and clinical outcomes.

**Findings:**

14 RCTs were included. There is a poor relationship between the dosage administered to children and symptom resolution, length of stay or need for mechanical ventilation. This study is limited due to its use of indirect evidence.

**Conclusion:**

The currently recommended dosage regimens may not represent the optimum safety and efficacy of intravenous aminophylline. There is a need to develop the evidence base correlating dosage with patient centered clinical outcomes, to improve prescribing practices.

## Introduction

Intravenous aminophylline can be used to manage exacerbations of asthma in children who do not respond to first line inhaled/nebulised therapy [1]. Accurate dosing is important, to ensure adequate asthma treatment, whilst reducing toxicity [2–4].

Aminophylline has a widely accepted therapeutic range of 10-20mg/l, which drives dosing decisions in children [1,5]. Current intravenous loading doses of between 5-6mg/kg are used to achieve levels within this range [6,7], although this is not regularly achieved in routine clinical practice [8–10]. Aiming for a target serum concentration of aminophylline is complicated by its high interindividual variation in clearance rates [11], the reasons for which are poorly understood [12–14]. It is not clear whether recommended adjustments of aminophylline dosage based on age, weight, and previous serum drug concentrations [5] optimise its efficacy and safety.

The treatment of acute asthma in children should be guided by evidence of improvement of clinically relevant outcomes [15,16]. A recent systematic review found no evidence to support improved efficacy of aminophylline once the serum concentration increases above the lower threshold of 10mg/L, nor increased toxicity at levels above 20mg/l [17]. As the current therapeutic range is a poor guide to efficacy, an alternative dosing strategy may improve the optimum efficacy and safety profile of aminophylline. This systematic review aims to evaluate the evidence for the optimum dosage strategy for aminophylline for children suffering an exacerbation of asthma.

## Methodology

### Study Design

We conducted a systematic review of studies utilizing intravenous theophyllines in the management of asthma exacerbations in children.

### Included studies

Few studies primarily investigate the optimum dosing strategies in children [18] Therefore, we used a systematic review technique which anticipates various study designs, similar to the methodology we recently used to examine the evidence around therapeutic ranges for aminophylline [17,19]. We decided a priori that the most relevant study type would be a comparison of randomised controlled trials (RCTs) comparing different dosing strategies and measuring clinically relevant outcomes, but we would also include RCTs evaluating the efficacy of intravenous theophyllines compared with placebo or other treatment, with subsequent analyses performed for each comparator drug, and observational studies.

We included studies that investigate the efficacy of intravenous theophyllines in children suffering an exacerbation of asthma if the dosing regimen was reported. We excluded studies performed in adults, those utilizing theophyllines for indications other than asthma or studies using non-intravenous routes.

### Outcomes

The pre-specified primary outcomes were time until resolution of symptoms, need for mechanical ventilation, and mortality. Secondary outcomes were the number of days until discharge criteria are met, number of days until actual discharge from hospital and adverse effects as defined and reported by authors.

### Identification of studies

The following search strategy was used to search MEDLINE, CINAHL and Web of Science in March 2016 with no date or language restrictions. The Cochrane Central Register of Controlled Trials was also searched for gray literature  asthma* AND (emerg* OR acute OR severe* OR intensive* OR exacerbation OR critical OR refractory OR hospitali*ed OR attack OR status) AND (aminophylline* OR intravenous theophylline* OR xanthin* OR methylxanthin*) AND (child* OR adolescent* OR infan* OR p*ediatric) 

Reviewer LC screened titles and abstracts, a second reviewer (IS or DH) checked the eligibility of abstracts after initial screening, and full studies included in the review. Reference lists were screened for other eligible studies.

### Data extraction and analysis

From each study we extracted the loading dose and/or maintenance dose of IV theophyllines administered, and whether subsequent doses were adjusted based on the results of therapeutic drug monitoring. The age range, number of participants and use of concomitant medications was also extracted.

#### Statistical analysis

We intended to conduct a quantitative synthesis by pooling studies utilizing similar dosing regimens using REVMAN (http://tech.cochrane.org/revman). Separate meta analyses for each dosing regimen used would allow for a quantitative comparison of effect size between studies using random effects modeling. If quantitate techniques were not possible due to methodological or reporting heterogeneity, or insufficient data, we planned for a descriptive analysis correlating pre-specified clinical outcomes to the dosing strategies used.

#### Assessment of risk of bias

The Cochrane Risk of Bias Tool was applied to each RCT to help determine the validity of results.

## Results

**Fig 1:**
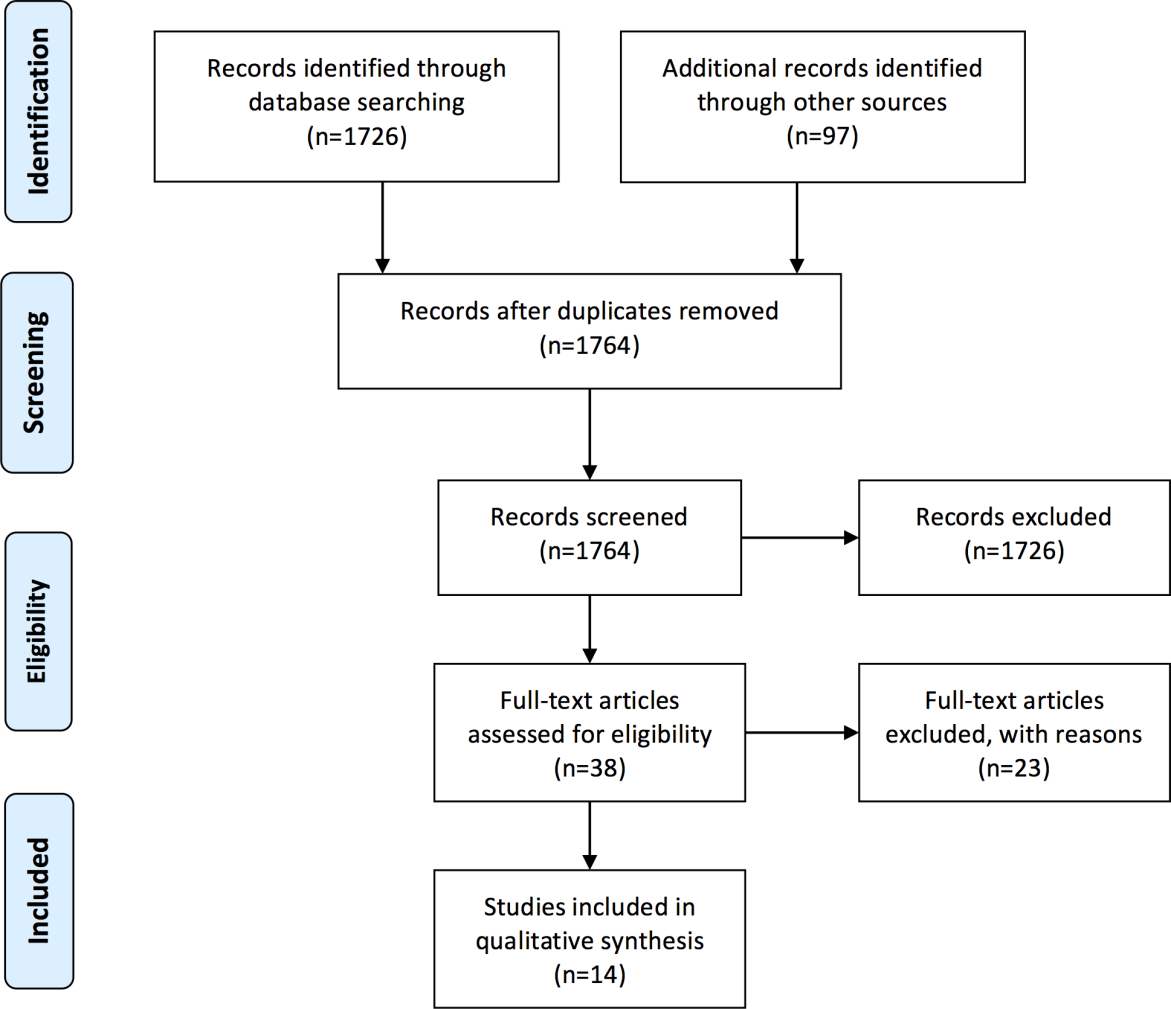
Search results.

Our search strategy returned 1764 studies, with 38 full text articles assessed for eligibility. We excluded 23 full text articles (S1 File) with the remaining 14 studies included in this systematic review (Fig 1).

No RCTs comparing dosing strategies for IV aminophylline in children suffering an exacerbation of asthma were identified. We therefore included 14 RCTs comparing aminophylline to placebo (n = 10) or β_**2**_ adrenergic agonists (n = 4).

### Risk of Bias of Included Studies

**Fig 2:**
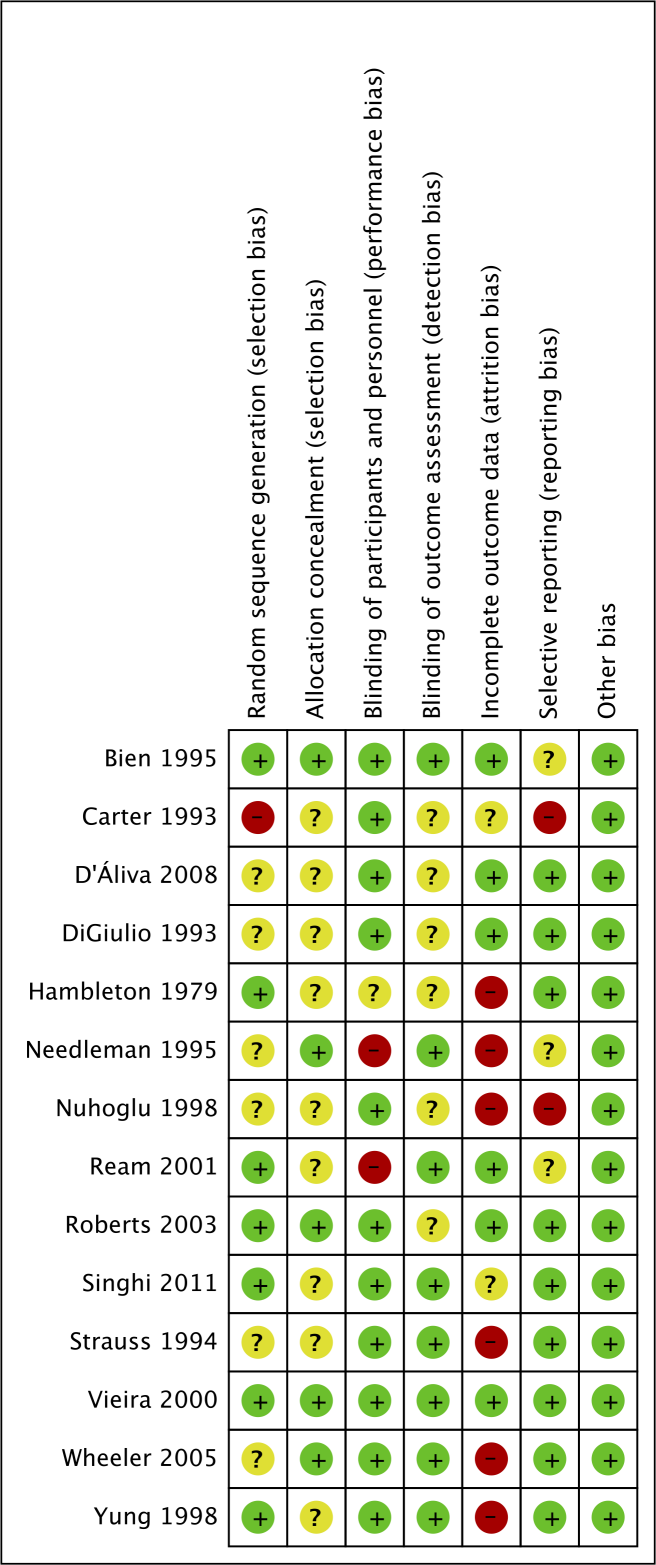
Results of assessment of risk of bias. Green–Low risk of bias, Red–High risk of bias, Amber–Unclear risk of bias.

The results from the Cochrane risk of bias assessment are shown in Fig 2. A high risk of selection bias was found in one study [20], performance bias was found in two studies [21,22], attrition bias in six studies [21,23–27] and reporting bias in two studies [23,28]. All other domains were found to have a low or unclear risk of bias.

### Clinical outcomes of studies using IV theophyllines for an acute exacerbation of asthma.

Studies were grouped based on whether they compared aminophylline to placebo or β_**2**_ adrenergic agonist. The loading doses, maintenance doses and clinical outcomes are shown in Tables 1 and 2.

**Table 1:** Results of RCTs comparing aminophylline to placebo *Loading doses altered based on the results of TDM. ASS asthma severity score, CAS/PI clinical asthma score/pulmonary index.

Author, sample size, (intervention vs control)	Loading (mg/kg)	Infusion (mg/kg/h)	Symptom resolution	Intubation	Discharge criteria	Actual Discharge	Adverse effects
**Yung 1998 [26] n = 163 (81 vs 82)**	10	0.7 to 1.1	Not reported	Not reported	Not reported	2.87 days vs. 2.69 days in p = 0.53	Higher rates of nausea and vomiting but not headache, irritability and tremor
**Needleman 1995 [21] n = 42 (22 vs 20)**	6–8	0.8 to 1	Change in ASS 6.96±1.65 vs 7.00±1.73 to 3.05±3.25 vs 2.38±2.19 [p = 0.482]	Not reported	52.3±32.3 hours vs 48.2±26.6 hours p = 0.654	Not measured	Not reported
**Strauss 1994 [29] n = 31 (14 vs 17)**	7	0.75 to 1.2	Not reported	Not reported	Not reported	2.58±1.5 days vs 2.33±1.3 days p>0.2	Higher rates of side effects in intervention vs aminophylline group 6/14 (43%) vs 1/17 (6%) in control p<0.05
**Ream 2001 [22] n = 47 (23 vs 24)**	7	0.5–0.8	Time to reach CAS<3 18.6±12.0 h vs 31.1±20.1 h; p = 0.0238	All subjects intubated before infusion	29.8±21.9h vs 36.4±25.9h p = 0.3774	4.7±1.3 days vs 5.1±1.8 days p = 0.432	No significant difference between intervention and control
**Nuhoglu 1998 [23], n = 38 (17 vs 19)**	6	0.8 to 1.0	Clinical asthma score at 24 hours in intervention vs placebo 2.05±1.61 vs 1.94±1.78 p = 0.8452	Not reported	Not reported	Not reported	NO significant difference between intervention and control
**DiGiulio 1993 [30] n = 29 (13 vs 16)**	6	0.85 to 1.0	Time to reach asthma score <2 in intervention vs control 30.4±16.n vs 27.0±10.3 p = 0.51	Not reported	30.4±16.8h vs 27.0±10.3h p = 0.51.	N/A	NO significant difference between intervention and control
**D’Ávila 2008 [31] n = 60 (30 vs 30)**	5	None	Not reported	Not reported	Not reported	43.2±3.30h vs 43.6±23.7h p = 0.95	Not reported
**Carter 1993 [28] n = 21 (12 vs 9)**	TDM*	0.8 to 1.0	Median CAS/PI at 36 hours 2.0 vs 2.0 p>0.05	Not reported	Not reported	3.5±2.5 days vs 3.0±1.5 days p =??	No significant difference between intervention and control
**Bien 1995 [32] n = 39 (19 vs 20)**	5*	0.9	CAS 24 hours in intervention vs control 2.0 vs 2.6 p>0.05	Not reported	Not reported	Not reported	Higher rates of nausea and emesis in theophylline group p≤0.05 but not insomnia p = 0.08
**Vieira 2000 [33] n = 43 (24 vs 19)**	6	1.2	Time to reach Wood-Downes score ≤ 2 12.5h vs 14.6h in p = 0.13	Not reported	Not reported	12.5h vs 14.6 h p = 0.13	No serious adverse events in either group

*Doses calculated based on the results of therapeutic drug monitoring. ASS asthma severity score, CAS/PI clinical asthma score/pulmonary index.

**Table 2:** Results of RCTs comparing aminophylline to β_2_ adrenergic agonist.

Author, sample size	Loading (mg/kg)	Infusion (mg/kg/h)	Resolution of symptoms	Intubation	Discharge criteria	Actual Discharge	Adverse effects
**Wheeler 2005 [25] n = 40 (Am n = 13, β**_**2**_ **n = 16, both n = 11)**	6.4	0.64 to 0.96	Time to reach CAS ≤3 24.2±12.1h vs 51.6±33.3h p<0.05	No patients required mechanical ventilation	Not measured	Not reported	NS in the median number of adverse effects, higher incidence of nausea in combined group
**Roberts 2003 [34] n = 44 (Am n = 26 β**_**2**_ **n = 18)**	5	0.9	Change in ASS over 2 hours -1.19±1.3 vs -1.11±1.7 p = 0.85	1/26 vs 2/18 in salbutamol p>0.05	Not measured	Time to discharge in aminophylline vs. salbutamol 57.3h±43.3 vs. 85.4h±56.0 p = 0.02	Adverse effects In aminophylline group vs. salbutamol 22.2% vs. 36% p = 0.50
**Singhi 2011 [35] n = 100 (Am n = 33, β**_**2**_ **n = 33, MgSO**_**4**_ **n = 34)**	5	0.9	number of participants with improvement in CAS at 1h ≥4 am, ter, 5 vs. 5 p = 0.002	Not reported	Not measured	Not reported	None in Mg group, 2 patients in terbutaline group had hypokalaemia and 9 in am group had nausea/vomiting
**Hambleton 1979 [27] n = 18**	4	0.6	Change in asthma score at 24 hours 4.5 vs 4.0 in p>0.05	Not reported	Not reported	Not reported	Higher rates of tachycardia in salbutamol group

Am aminophylline, β_2_ beta 2 agonist, ASS asthma severity score, MgSO_4_ magnesium sulphate CAS/PI clinical asthma score/pulmonary index. Sizes of intervention vs. control groups were not reported in Hambleton 1979

### Doses of aminophylline given

The doses given to children across RCTs utilizing IV aminophylline for an acute exacerbation of asthma in children is highly variable. All but one study [31] prescribes aminophylline as a loading dose followed by an infusion. All studies calculate doses based on the weight of individual participants. Loading doses range from 4-10mg/kg and infusion rates range from 0.5–1.2mg/kg/hr.

Age was factored into dosing strategies of aminophylline in eight studies. Age influenced both the loading dose and the infusion rate given in one study [21], with the remaining seven studies using age adjusted maintenance doses only [22–26,30,31]. In most studies, younger patients received higher doses of IV aminophylline.

The results of therapeutic drug monitoring (TDM) factored into aminophylline dosage calculations in the majority of studies. Infusion rates were adjusted based to keep serum theophylline levels within a predefined range in nine studies [21,23–26,28,30,32,34] In two studies [28,32] serum theophylline levels were factored into loading dose calculations.

### Quantitative Synthesis

**Fig 3:**
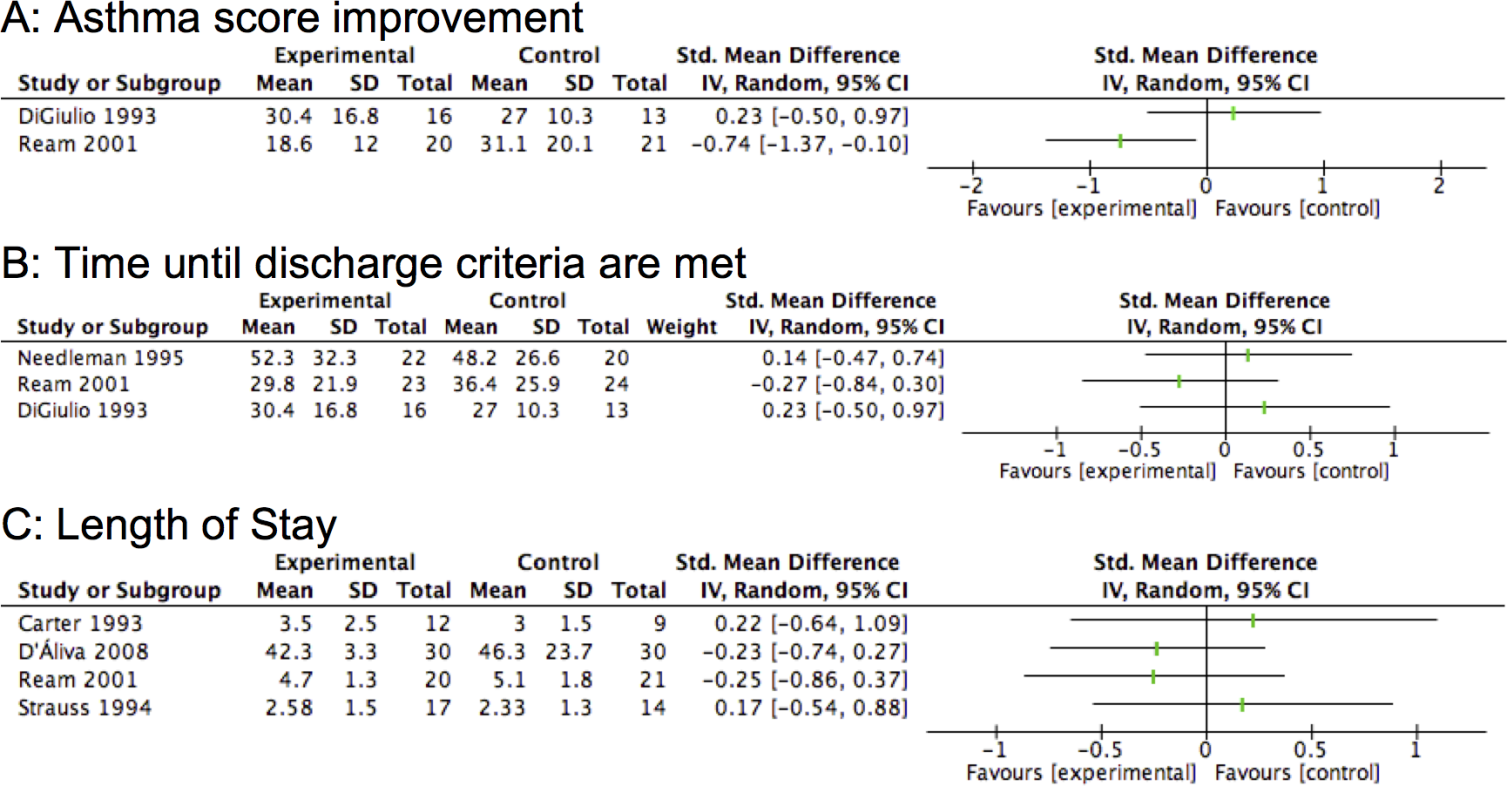
Quantitative synthesis of studies comparing aminophylline to placebo. Dose Regimens: Digiulio 1993 6mg/kg bolus 0.8–1.0mg/kg/h infusion, Ream 2001 7mg/kg bolus 0.5–0.8mg/kg/h infusion, Needleman 1995 6-8mg/kg bolus 0.8–1.0mg/kg/h infusion, Carter 1993 loading dose calculated on TDM infusion 0.8–1.0mg/kg/h, D’Aliva 2008 2 x 5mg/kg bolus, Strauss 1994 7mg/kg/h bolus 0.75 to 1.2 infusion

Quantitative synthesis was possible for studies comparing aminophylline to placebo in the domains of resolution of symptoms, time until discharge criteria are met, and length of stay (Fig 3). Meta analysis was not possible for studies comparing aminophylline to β_2_ agonists due to incomplete outcome reporting.

### Primary Outcomes

#### 1) Time until resolution of symptoms

Symptom resolution was reported in seven studies comparing aminophylline to placebo. Asthma score after a given time was reported in four studies [21,23,28,32] and time to reach a predefined asthma score was reported in three studies [22,30,33]. Adequate reporting in two studies allowed quantitative data synthesis (Fig 3). All four studies comparing aminophylline to β_**2**_ adrenergic agonist reported symptom resolution as an outcome. This was reported as time to reach a predefined asthma score in one [36], change in asthma score in two [27,34] and the proportion of patients in each group achieving a low asthma score in the remaining score [37].

There appeared to be no discernible relationship between aminophylline dosage and improvement in symptoms. Although one study reported quicker improvement in asthma score with a loading dose of 7mg/kg followed by an infusion of 0.5–0.65mg/kg/hr (time to reach CAS≤3 in aminophylline vs placebo group 18.6±12.0h, n = 23 vs 31.1±20.1h, n = 24 [p = 0.0238]) [22], this finding is not replicated in studies using similar doses [21,30]. Intravenous aminophylline at any dose was equally effective when compared to β_**2**_ adrenergic agonist at improving symptoms.

#### 2) Need for Mechanical ventilation

No studies comparing aminophylline to placebo assessed effect of IV aminophylline against placebo, in non-intubated children, on the subsequent need for mechanical ventilation. One study comparing a 5mg/kg loading dose followed by an infusion of 0.9mg/kg/h to β_**2**_ adrenergic agonist, found a that one subject in the aminophylline group and 2 in the β_**2**_ adrenergic agonist group required mechanical ventilation [p>0.05] [34] however it is not possible to compare this finding with other doses given.

#### 3) Mortality

There were no reported deaths in any study

### Secondary outcomes

#### 1) Time until discharge criteria are met

Time until discharge criteria are met was reported in three studies comparing aminophylline to placebo, one using a 7mg/kg loading dose followed by an infusion of 0.5–0.8mg/kg/hr [22] and one using a 6mg/kg loading dose followed by an infusion rate of 0.85–1.0mg/kg/hr [30], and one adjusting loading and maintenance doses based on age [21]. No studies reported a significant improvement in time until discharge criteria are met with the use of intravenous aminophylline at any dose. One study reported an improvement in the very small subset of patients who were intubated prior to enrollment 74.8±15.4, n = 3 in theophylline group vs 189.3±59.8, n = 3 in control p = 0.0325 [22]. Three studies were included in quantitative data synthesis (Fig 3).

#### 2) Actual Discharge

Length of stay was reported in five studies comparing aminophylline to placebo [22,24,28,31,38] and one study comparing aminophylline to β_**2**_ adrenergic agonist [34]. Four studies reported the number of days spent in hospital [22,24,28,38] one study reported the number of hours spent in the paediatric emergency room, [31] and one study reported the number of hours spent in hospital [34]. No statistically significant difference was observed in shortening hospital length of stay at any dose of aminophylline when compared with placebo. A loading dose of 5mg/kg followed by an infusion of 0.9mg/kg/h was shown to significantly shorten hospital stay compared with β_**2**_ adrenergic agonist (57.3h±43.3, n = 26 vs 85.4h±56.0, n = 18 [p = 0.02]) [34]. Four studies were included in quantitative data synthesis (Fig 3).

#### 3) Adverse effects

Adverse effects were compared in six studies comparing aminophylline to placebo [23,24,28,30,32,38] and no studies comparing aminophylline to β_**2**_ adrenergic agonist.

There appears to be a higher rate of adverse effects in participants receiving higher loading doses. A significantly higher rate of adverse effects was reported in two studies using a loading dose of 10mg/kg and 7mg/kg [24,26] but not in studies using loading doses between 5-6mg/kg [22,23,30,33]. One study reported a higher rate of adverse effects in subjects receiving a loading dose calculated using 500ml/kg X change in serum level formula [32].

## Discussion

There is a lack of evidence on which to determine to most effective and safe intravenous dosage of aminophylline for children suffering an acute exacerbation of asthma. There is weak evidence to suggest that loading doses above 7mg/kg result in a higher rate of nausea and vomiting. There is no evidence to indicate that adjustment of dose based on age or serum theophylline levels increases the efficacy or safety of IV aminophylline.

No RCTs have directly compared dosing strategies for aminophylline when used for acute asthma exacerbations in children. The indirect evidence from RCTs comparing aminophylline with placebo demonstrates no clear relationship between dosage regimen, which varies across studies, and clinical efficacy and safety. The majority of dosing strategies aim to achieve serum theophylline levels within a predefined range, but this did not translate into clinical improvement.

Forming dosing recommendations for IV aminophylline in children is complex as the drug is used in a wide age range of children with highly variable pharmacokinetic properties. Although efforts to account for this variability are reflected in dosing adjustments made for age, weight and previous theophylline levels, it is unclear whether these adjustments play a significant role in improving the clinical outcomes of children with acute asthma. Dosing strategies based on evidence of clinical improvement are an important factor when comparing the efficacy of intravenous bronchodilators for the treatment of childhood asthma. There is a need for research linking the pharmacokinetic knowledge of theophylline, with clinically relevant outcomes in acute asthma in children.

As no RCTs compare different aminophylline dosage in the treatment of childhood asthma exacerbations, this review is hindered by its use of indirect evidence. Our quantitative analyses are limited by the small sample sizes, varied dosing strategies and inconsistent outcome reporting across studies. This meta analysis is not able to provide additional information to improve prescribing practices or guide future trial design. Furthermore, this review included studies spanning a 32 year time period and intravenous theophylline and aminophylline (theophylline with ethyldiamine) were considered together.

The optimal dosing of IV aminophylline in acute childhood asthma requires accurate assessment of its efficacy and adverse effects. A recent survey of paediatric emergency departments in the British Isles has shown that over 95% of children receive the same loading dose (5mg/kg) [39]. Though dosage guidelines are followed, the clinical outcomes of this strategy are not clear. Prior to conducting RCTs comparing doses, we believe that it is important to establish the clinical effectiveness of the currently recommended dose, to establish the level of clinical improvement seen using validated asthma scores in children, and provide adverse effect data. This will allow the paediatric asthma community to determine if the current benefit:risk ratio of IV aminophylline is satisfactory, and whether RCTs with alternate doses (to improve efficacy using higher doses, or avoid adverse effects using lower doses) are appropriate.

## Conclusion

The currently recommended dosage strategy of intravenous aminophylline may not represent the optimum safety and efficacy profile of the drug in childhood asthma exacerbations. There is poor evidence that dosage adjustments based on age weight and previous serum theophylline levels improve asthma outcomes in children. An investigation correlating dosage to clinically relevant outcomes is needed to develop studies aiming to improve prescribing practices of intravenous aminophylline.

## Supporting Information

S1 File Excluded Studies after reading full text. (DOCX)

S2 File PRISMA Checklist. (DOC)
