# Supplementary material for: Aminophylline Dosage In Asthma Exacerbations in Children: A Systematic Review
Source: PLoS One. 2016 Aug 2;11(8):e0159965. doi: 10.1371/journal.pone.0159965 (PMC4970720; doi:10.1371/journal.pone.0159965)
Supplement: S1 File — (DOCX) [file pone.0159965.s001.docx]

| **Title of Study** | **Reason** | **Ref.** |
| --- | --- | --- |
| Ketamine versus aminophylline for status asthmatic in children: A randomized, controlled trial | No other studies used ketamine as a comparator drug. Unable to compare across studies | (1) |
| Efficacy and Safety of Intravenous Aminophylline in Children with Acute Exacerbation of Asthma: A Multicenter Randomized Trial | Supplement article, no full text available | (2) |
| Theophyllines and magnesium sulfate in severe asthmatic crisis [Teofilinas y sulfato de magnesio en la crisis asmática grave] | Not a randomised controlled trial | (3) |
| Efficacy and safety of intravenous theophylline administration for treatment of mild acute exacerbation of bronchial asthma | Adult study | (4) |
| Prospective multicenter study of relapse after treatment for acute asthma among children presenting to the emergency department | Cohort study | (5) |
| Efficacy of intravenously administered theophylline in children with moderate asthma attacks [ORTA SIDDETTE AKUT ASTMA ATAGINDA INTRAVENOZ TEOFILIN TEDAVISININ ETKISI] | Not an RCT | (6) |
| Aminophylline in the hospital treatment of children wih acute asthma | Editorial article | (7) |
| Effect of adding aminophylline infusion to nebulised salbutamol in severe acute asthma | Adult study | (8) |
| Intravenous theophylline after beta 2-agonist treatment in severe acute asthma. Effect on patients who are not pre-treated with theophylline. | Adult study | (9) |
| Comparison between theophylline and an adenosine non-blocking xanthine in acute asthma | Adult study | (10) |
| Aminophylline loading in asthmatic patients: A protocol trial | Includes adults and children, not treated separately in analysis | (11) |
| Treatment of acute asthma. Is combination therapy with sympathomimetics and methylxanthines indicated? | Adult study | (12) |
| Clinical and pharmacokinetic studies on theophylline treatment for childhood asthma | Not an RCT | (13) |
| Aminophylline increases the toxicity but not the efficacy of an inhaled beta-adrenergic agonist in the treatment of acute exacerbations of asthma | Supplement article only, full text not available | (14) |
| A controlled trial of the use of single versus combined-drug therapy in the treatment of acute episodes of asthma | Adult study | (15) |
| Intravenous aminophylline therapy for asthma. A comparison of two methods of administration in children. | Not a randomised controlled trial | (16) |
| Emergency therapy of asthma: comparison of the acute effects of parenteral and inhaled sympathomimetics and infused aminophylline | Adult study | (17) |
| Aminophylline, salbutamol and combined intravenous infusions in acute severe asthma. | Adult study | (18) |
| Cardiac dysrhythmias during the treatment of acute asthma. A comparison of two treatment regimens by a double blind protocol | Adult study | (19) |
| Bronchodilator effects of terbutaline and aminophylline alone and in combination in asthmatic patients. | Adult study | (20) |
| A controlled trial of intravenous salbutamol and aminophylline in acute asthma. | Adult study | (21) |
| Double-Blind Trial of Aminophylline in Status Asthmaticus. | Did not report dose | (22) |
| Emergency room treatment of asthma ^☆^: Relationships among therapeutic combinations, severity of obstruction and time course of response | Adult study | (23) |

**Full text articles excluded with reasons**

1. Tiwari A, Guglani V, Jat KR. Ketamine versus aminophylline for status asthmatic in children: A randomized, controlled trial. European Respiratory Journal 2014;44(Suppl 58):281.

2. Nagao M, Katsunuma T, Kim CK, Fujisawa T. Efficacy and safety of intravenous aminophylline in children with acute exacerbation of asthma: A multicenter randomized trial. The Journal of Allergy and Clinical Immunology 2007, Jan;119(1):S2.

3. Alcaraz AJ, Panadero E, Santiago MJ. Theophyllines and magnesium sulfate in severe asthmatic crisis [teofilinas y sulfato de magnesio en la crisis asmática grave]. Anales De Pediatria Continuada 2006;4(4):237-40.

4. Yamauchi KA, Kobayashi HA, Tanifuji YA, Yoshida TA, Pian HDA, Inoue HAB. Efficacy and safety of intravenous theophylline administration for treatment of mild acute exacerbation of bronchial asthma. Respirology 2005;10(4):491-6.

5. Emerman CLABCD, Cydulka RKABCD, Crain EFABCD, Rowe BHABCD, Radeos MSABCD, Camargo Jr. CAABCD. Prospective multicenter study of relapse after treatment for acute asthma among children presenting to the emergency department. J Pediatr 2001, Mar;138(3):318-24.

6. Tomac N, Saraclar Y, Tuncer A, Adilioglu G, Cengizlier R. Efficacy of intravenously administered theophylline in children with moderate asthma attacks [ORTA SIDDETTE AKUT ASTMA ATAGINDA INTRAVENOZ TEOFILIN TEDAVISININ ETKISI]. Cocuk Sagligi Ve Hastaliklari Dergisi 1996;39(3):423-30.

7. McKenzie SA. Aminophylline in the hospital treatment of children wih acute asthma. Br Med J 1994;308(6941):1384-5.

8. Zainudin BMZ, Ismail O, Yusoff K. Effect of adding aminophylline infusion to nebulised salbutamol in severe acute asthma. Thorax 1994;49(3):267-9.

9. Janson C, Boman G. Intravenous theophylline after beta 2-agonist treatment in severe acute asthma. Effect on patients who are not pre-treated with theophylline. Upsala Journal of Medical Sciences 1992;97(2):149-55.

10. Vilsvik JS, Persson CG, Amundsen T, Brenna E, Naustdal T, Syvertsen U, et al. Comparison between theophylline and an adenosine non-blocking xanthine in acute asthma. European Respiratory Journal 1990;3(1):27-32.

11. Stine RJA, Marcus RHA, Parvin CAB. Aminophylline loading in asthmatic patients: A protocol trial. Ann Emerg Med 1989;18(6):640-6.

12. Fanta CH, Rossing TH, McFadden ER. Treatment of acute asthma. Is combination therapy with sympathomimetics and methylxanthines indicated? The American Journal of Medicine 1986, Jan;80(1):5-10.

13. Seki M. Clinical and pharmacokinetic studies on theophylline treatment for childhood asthma. Medical Journal of Kobe University 1986;47(1):1-9.

14. Siegel D, Sheppard D, Gelb A, Weinberg PF. Aminophylline increases the toxicity but not the efficacy of an inhaled beta-adrenergic agonist in the treatment of acute exacerbations of asthma. Am Rev Respir Dis 1985, Aug;132(2):283-6.

15. Rossing TH, Fanta CH, McFadden Jr. ER. A controlled trial of the use of single versus combined-drug therapy in the treatment of acute episodes of asthma. American Review of Respiratory Disease 1981;123(2):190-4.

16. Goldberg P, Leffert F, Gonzalez M, Gogenola L, Zerbe GO. Intravenous aminophylline therapy for asthma. A comparison of two methods of administration in children. Am J Dis Child 1980;134(6):596-9.

17. Rossing TH, Fanta CH, Goldstein DH, Snapper JR, McFadden Jr. ER. Emergency therapy of asthma: Comparison of the acute effects of parenteral and inhaled sympathomimetics and infused aminophylline. American Review of Respiratory Disease 1980;122(3):365-71.

18. Evans WV, Monie RD, Crimmins J, Seaton A. Aminophylline, salbutamol and combined intravenous infusions in acute severe asthma. Br J Dis Chest 1980, Oct;74(4):385-9.

19. Josephson GW, Kennedy HL, MacKenzie EJ, Gibson G. Cardiac dysrhythmias during the treatment of acute asthma. A comparison of two treatment regimens by a double blind protocol. Chest 1980;78(3):429-35.

20. Wolfe JD, Tashkin DP, Calvarese B, Simmons M. Bronchodilator effects of terbutaline and aminophylline alone and in combination in asthmatic patients. N Engl J Med 1978, Feb 16;298(7):363-7.

21. Tribe AE, Wong RM, Robinson JS. A controlled trial of intravenous salbutamol and aminophylline in acute asthma. The Medical Journal of Australia 1976;2(20):749-52.

22. William P, Warren B, Stanley S. Double-Blind trial of aminophylline in status asthmaticus. Pediatrics 1971, Oct;48(4):642.

23. Fanta C, Rossing T, Mcfadden E. **Emergency room treatment of asthma** ^☆^**: Relationships among therapeutic combinations, severity of obstruction and time course of response.** American Journal of Medicine 1982;72(3):416-22.
